# Position statement GMA Comittee – “Interprofessional Education for the Health Care Professions”

**DOI:** 10.3205/zma000964

**Published:** 2015-05-13

**Authors:** Ursula Walkenhorst, Cornelia Mahler, Regina Aistleithner, Eckhart G. Hahn, Sylvia Kaap-Fröhlich, Sven Karstens, Karin Reiber, Beate Stock-Schröer, Beat Sottas

**Affiliations:** 1 Universität Osnabrück, FB Humanwissenschaften, Osnabrück, Deutschland; 2 Universitätsklinikum Heidelberg, Abt. Allgemeinmedizin und Versorgungsforschung, Heidelberg, Deutschland; 3 Gesundheit Österreich GmbH, Gesundheitsberufe, Wien, Österreich; 4 Gesellschaft für Berufliche Fortbildung, Forschung und Entwicklung e.V. an der Medizinischen Klinik 1, Universitätsklinikum Erlangen, Erlangen, Deutschland; 5 Universität Zürich, Medizinische Fakultät, Zürich, Schweiz; 6 Hochschule Esslingen, Fakultät Soziale Arbeit, Gesundheit und Pflege, Esslingen, Deutschland; 7 Karl und Veronica Carstens-Stiftung, Aus- und Weiterbildung, Essen, Deutschland; 8 Stiftung Careum, Stiftungsrat, Zürich, Schweiz

## Preface

The discussion about new forms of cooperation and competencies in the health professions need not primarily take place from the perspective of the occupational groups, but rather on the basis of future expectations of the healthcare system – meaning from the perspective of the patients [1].

The Committee for Interprofessional Education in the Health Professions was established in 2011 as a sub-committee of the *Gesellschaft für medizinische Ausbildung* (GMA) and is composed of German, Austrian and Swiss experts in the fields of medicine, nursing, and the therapeutic and diagnostic health professions. The committee was founded with the task of critically reviewing current Interprofessional Education programs in academia among the health professions and making recommendations for Interprofessional Education in the form of a position statement. This is to provide both theoretical and practical support regarding further developments to integrate interprofessional approaches into education for the health professions. The need to systematically address this topic has arisen from previous observations about the necessity for collaboration between the health professions [2], [3], [4], as well as knowledge gained about the effects of cooperation in terms of all stakeholders: patients, the general population, and members of the health professions. 

The committee first looked at the initial occupational training in selected health professions^1^ (within the German practice context), which have been undergoing academization in recent years and differ in this respect from the long-established academic field of medicine. The recommendations formulated in this position statement concerning Interprofessional Education are not directed solely at a particular ccupational group, but are intended to address all health professional groups that directly contribute to patient care. Presently, with the exception of medicine, a situation exists in which the majority of initial training programs for the health professions are still embedded in the vocational education system. This starting point has been given special attention by the committee members, particularly in the discussions with representatives from medicine. From the perspective of healthcare policy [5], the current developments towards the academization of health professions also have implications for future educational governance and thus for the future development of healthcare with all professional groups.

We wish to extend our gratitude to all the members of the GMA Committee for Interprofessional Education in the Health Professions and to all those who participated in the discussions that took place in various workshops and committee meetings and who provided important stimuli and suggestions for drafting this position statement.

## Introduction

Interprofessional Education at undergraduate level has the aim of laying the groundwork for later interprofessional collaboration among those working in the field of healthcare and, as a result, of contributing to reliable health care outcomes for patient-centred care. Within the scope of this committee, the topic was approached not only from the necessary perspective of healthcare provision, but also from an educational standpoint and the recommendations formulated here focus on the educational and pedagogical perspectives. This position statement can be used as a stimulus for further discussions that must be held in the areas of health and education. A systematic perspective connecting both areas is absolutely necessary for implementing these recommendations.

In past years, the committee has looked closely at the national situations in the German-speaking countries^2^ concerning education in the health professions and also at international experiences regarding collaboration among the health professions. An inventory of national and international approaches and concepts in Interprofessional Education was compiled, and empirical studies investigating the effectiveness of interprofessional cooperation and interprofessional learning were reviewed and analyzed. The criteria for dealing with the structures and content indentified in the studies as being pre-requisite for successful implementation of Interprofessional Education, along with the detrimental factors, were compiled and form the basis of this position statement.

Firstly, the initial status, cooperative efforts, and national and international examples are presented, and the current state of research is summarized. Then, the committee’s recommendations are given for the corresponding implementation strategies to improve Interprofessional Education.

## Starting point

Healthcare today and that of the future are confronted with demographic and epidemiological challenges. Ensuring and developing healthcare can only be guaranteed by excellent, interprofessional collaboration between all existing health professions, and any that may emerge. In preparing for these challenges in providing healthcare, numerous implications arise for education. In addition, human resources and the focus on economic factors means that practice will continue to take place within a context of tight resources.

Parallel to this, the academization and professionalization [6], [7] of the health professions in the German-speaking countries are increasingly contributing to better qualifications for these occupations in healthcare and, based on the establishment of a broader scientific basis and research, are bringing to the healthcare system new insights about different aspects of healthcare needs and new approaches for healthcare services. Organizing collaboration with medical colleagues now plays a major role in this development, as further academization of the health professions needs to take place in close alliance with the representatives from the field of medicine. In what form the necessary interprofessional cooperation can and must be organized and experienced cannot be completely answered at present and requires further refinement and discussion.

Sequences and structures in interprofessional educational programs, at both the level of vocational training and university study, are currently rare for all health professions, but are gaining increased importance as bachelor degree programs in nursing and the therapeutic professions are being established. In medical education, initial approaches for interprofessional learning with the other health professions are emerging, particularly as new educational concepts and model curricula. At present, effective collaboration between the health professions mostly depends on the motivation of individual efforts by persons and small teams. In order to expand this form of cooperation step-by-step into sustainable and forward-looking structures, it will be necessary to have suitable concepts. These, in turn, require an essential dialogue between the health professions and an anchoring within educational programs and their legal regulatory frameworks. Educational institutions should take up this topic within the context of existing structures and guidelines to do justice to their future-oriented mission of contributing to better healthcare. At the same time, it is important to work on legislation that ensures the qualifications for interprofessional work are covered by educational curricula.

### Developmental state of Interprofessional Education concepts in selected European countries

The status of Interprofessional Education varies greatly among reviewed countries due to the different locations and regulation of educational programs. Selected examples from some European countries are described in the following:

#### a) Germany

In Germany, the socialization of the medical, nursing, therapeutic and diagnostic health professions during training and university study takes place for the most part separately from each other. The following briefly presents initial model projects in interprofessional education.

The University for Health (*Hochschule für Gesundheit*) in Bochum has for the first time integrated interprofessional curricula units over the entire course of the degree programs in occupational therapy, physiotherapy, speech and language therapy, midwifery and nursing [http://www.hs-gesundheit.de/de/thema/die-hochschule/, last verified on 2 March 2015] and also integrates learning with students at the medical school of the *Ruhr Universität Bochum* (see below) in projects. At the Medical Faculty of Heidelberg University, a Bachelor degree program in Interprofessional Healthcare was established in 2011, in which nine different health professions complete an academic degree in parallel to their vocational training (i.e. geriatric nursing, general nursing, pediatric nursing, midwifery, speech and language therapy, physiotherapy, orthoptics, medical laboratory technicians andradiography ) [8]. Within this interprofessional degree, some modular learning takes place with medical students enrolled at the same faculty. Subjects such as team communication [9], Health Care English, and medical error management/error culture are integrated into both curricula .

In recent years, the first model projects between universities of applied sciences and university medical schools have also been initiated, in which students study together. For example, as part of an interprofessional model project between the Medical Faculty of the *Ruhr-Universität Bochum* and the *Hochschule für Gesundheit*, students of medicine, nursing, therapy and midwifery work together on a case [10]. With its interprofessional learning program in the health professions, *Operation Team – Interprofessionelles Lernen in den Gesundheitsberufen*, the Robert Bosch Foundation supports current model projects that develop, conduct and evaluate educational courses involving medical schools and various other health professions. The first publications on this are expected to appear in 2015.

##### b) Austria

Educational programs in Austria leading to university degrees in the health professions are currently monoprofessional. Since the 2005 health reform, legislators, in response to healthcare needs, have been giving increased consideration to multi-professional approaches not only in terms of healthcare provision, but also education. In respect to educational programs in the health professions, this requirement is particularly reflected in the competence-oriented university rules and regulations for nursing, midwifery and technical lab services (biomedical analysis, dietetics, occupational therapy, speech and language therapy, orthoptics, physiotherapy, and radiography). Approaches to Interprofessional Education are facilitated by programs in multiple health professions at the same institution. These are especially present in the form of advanced training at the Master’s degree level. Currently, the health reform of 2013, initiated by the federal government, states and social insurance providers, aims for a paradigm change that will lead to appropriate modifications in the educational programs for the health professions. Taking the “Health in all Policies” approach and the basic health objectives into consideration, the focus is on health promotion and preventive healthcare. A concept for graduated care is meant to ensure needs-based, effective and efficient care which, in turn, guarantees the high quality of care. Interdisciplinary, multiprofessional, and integrated forms of care are intended at all levels of care. At present, the necessary structural, organizational and financial conditions are being developed for the individual levels of care. Based on this, the roles and competencies of the health professions involved will be defined. Following this, any required amendments to the legislation governing education will be undertaken. A current project on educational reform in nursing, as well as one dealing with medical study, will take these aspects into account, along with the modifications to the EU Directive on the recognition of professional qualifications (Directive 2013/55/EU). By 2016, the first results regarding changes to education in response to the health reform should be available and implemented.

##### c) Switzerland

The awareness of Interprofessional Education (Interprofessional Education, IPE) is high in Switzerland and willingness to take concrete action has been signaled and steps for implementation have been initiated. In doing this, differentiation has been made between two levels: on the level of political strategy, the governing Swiss Federal Council has given impetus for interprofessional approaches to healthcare and reinforced the health policy framework Health 2020 with a strategy paper on Interprofessional Education up to the level of an educational reference framework [http://www.gesundheit2020.ch, last verified on 1 March 2015]. The forces driving policy-makers to set this course are the future challenges of providing appropriate healthcare, particularly regarding access to care and maintaining an excellent quality of care. As a consequence of the increasing shift from inpatient to outpatient care, expectations are being placed on innovative developments in the area of Advanced Practice.

At the level of the educational institutions many separate interprofessional projects can be indentified. This development is promoted by general objectives in the law governing education and competence-based curricula for health professions that are implemented at the medical schools, universities of applied science, and institutes of higher vocational education. As a result of the academization of the health professions and the consistent implementation of the Bologna Reform, comparable curricular structures have also been created that simplify the introduction of interprofessional programs at the formal level. Competencies corresponding to the role of the (interprofessional) collaborator in the CanMeds role models [11] are not only described in the competencies expected of the non-university health professions, but also in the Swiss catalogue of learning objectives SCLO [12] and the catalogue of learning objectives for advanced medical training programs [http://www.fmh.ch/files/pdf8/allg_lz_d.pdf, last verified on 2 March 2014]. The competencies for the health professions studied at universities of applied science focus more strongly on interprofessional skills between these health professions.

Interprofessional Education and interprofessional training vary nevertheless between the educational institutions and in practice. An overview of the interprofessional activities in education at Swiss universities and universities of applied science can be found in the report by the topic group for Interprofessionalism at the Swiss Federal Office of Public Health [http://www.bag.admin.ch/themen/berufe/11724/14204/index.html?lang=de, last verified on 1 March 2015]. 

Although there may be a bold consensus in this atmosphere of optimistic progress (Swiss Academy of Medical Sciences, see below), the jury is still out in respect to the results of this transitional phase – whereby the dynamic that has been created increases the pressure on politics, healthcare, and education.

A political mandate issued by the federal government and cantons is being striven for so that IPE can be pushed in a targeted manner as preparation and qualification for interprofessional practice at the national level. As this takes place, attention must be turned not only toward patient-oriented functions, but also toward topics, such as management, financing, and logistics. Anchoring all this in the organization of curricula must also be addressed.

The work on the charter *Zusammenarbeit der Gesundheitsberufe* (Collaboration among the Health Professions) of the Swiss Academy of Medical Sciences is to be viewed positively, in that reference is also made to Interprofessional Education and advanced training [13]. This charter was made public in 2014 and then widely disseminated.

##### d) Scandinavia

In 1986, the medical school at Sweden’s Linköping University added a 12-week interprofessional learning phase for medical students, nurses, physiotherapists, occupational therapists, speech and language therapists, and medical biologists to its curricula. The interprofessional learning is divided into three sequential stages and integrated into different phases of study. Already at the beginning of university study, the students learn together in a seven-week joint module on the principles of health and health promotion and on health and socio-political issues. This lays the foundation for problem-based learning. After two and a half years, the students come together again for the second part in a two-week topic-related course that focuses on the development of complementary professional skills and awareness of the different professional identities. In the third part, the students participate together on interprofessional teams in a two-week internship, during which they are responsible for giving patient care in real surroundings on a training ward. Following an introductory phase, the students organize and assign the necessary tasks and responsibilities on their own. The interprofessional learning is then completed with a reflection on the learning experience [http://www.hu.liu.se, last verified on 2 March 2015].

##### e) England

Another example of how interprofessional units are anchored into the curriculum can be seen at the University of Southampton in England, where since 2000 internet-based common learning has taken place. This encompasses two interprofessional units, in which each year around 3,500 students from eleven health professions and the field of social work exchange information with each other. Not only is joint learning content imparted to the students, but they also receive training in interprofessional skills. The first curricular unit serves as an introduction to the concept of collaborative learning and teamwork, as well as the IT methods for the subsequent internet-based modules. In the second unit, students then have the opportunity to jointly apply teamwork and negotiating skills in an interprofessional context during an audit [14].

## Current state of research

After searching for relevant literature in professional journals and databases, a total of 58 publications were found on the topic of Interprofessional Education (IPE) and Interprofessional Collaboration (IPC) (as of spring 2013). A meta-analysis in the form of a Cochrane Review [15], [16] exists for each of the two topics, along with diverse overviews. These describe the various models of common learning and collaboration, as well as cover the evaluations of individual projects. Most studies were carried out in Canada, the U.S., Sweden or England. There are only a few publications covering Germany, Austria and Switzerland. Sweden (Linköping University) [17] and Canada (University of British Columbia) [18] are at the fore not only for IPE, but also IPC.

Results of studies show that Interprofessional Education (IPE) in joint educational projects at universities leads to better understanding for the other professions and that the satisfaction with one’s own profession increases [19]. It is important to keep the learning groups as small and heterogeneous as possible and to have the teaching focus strongly on practice. Coordinating the different curricula proved difficult in the models described. Among the professional groups analyzed were dentists, physicians, nursing staff, pharmacists, social workers, occupational therapists, physiotherapists, midwives and radiographers. In the few and very heterogeneous studies, which were included in the Cochrane Reviews by Reeves et al. [15] and Zwarenstein et al. [16], it has not been possible to recognize any effects that can be generalized regarding modified behavior when providing healthcare or on patient outcomes, despite indications of positive results arising from interprofessional learning and interprofessional collaboration.

The studies found on Interprofessional Collaboration (IPC) also present different assessments. All of the health professions addressed in these studies agree on the necessity of collaboration, but these same professions differ on how to implement it. Satisfaction in practice also differs according to profession. Nevertheless, individual studies prove shorter hospital stays for patients and a reduced need for medication if interprofessional cooperation takes place in teams in the form of regular coordination and case conferencing [20], [21]. Clear structures in respect to responsibilities in the therapy setting also appear to be important [22].

The conclusion drawn by almost all of the studies is that it is the patient who ultimately profits from improved cooperation among and coordination of all the professions in the healthcare system. However, this hypothesis must be further demonstrated in the form of convincing empirical studies. Such collaboration and better understanding of other professional groups can be best realized during education in the form of interprofessional curricular sequences. In addition, virtually all of the overviews see a great need for methodically well-founded research in this area.

## Recommendations

The recommendations based on the analysis of the current situation, the examples from European countries, and the outline of the current state of research are formulated from the perspective of the field of education and pedagogy, even if other viewpoints (healthcare, healthcare policy, occupation policy) do indeed seem possible. One reason for the focus on the educational perspective stems from the task of the committee and interests of the GMA, which is to improve education in medicine and the other health professions.

After extensive research, joint discussions and analysis of the data, the committee makes the following recommendations, which will require further definition for application and appropriate adaptation for the various institutions.

### 1. Concepts for Interprofessional Education

The development of Interprofessional Education programs in the German-speaking countries has gained in significance, but there is still a lack of feasible and sustainable concepts that support educational institutions in developing their own interprofessional approaches and programs, as well as the necessary structures for this.

The committee recommends:

The development of an overall concept for interprofessional competence that is oriented toward all educational and training programs in the health professions in Germany, Austria and Switzerland. This concept will be drawn up by an interprofessional expert panel of representatives from the three countries, similar as the “Core competencies for interprofessional collaborative practice” [23]. To prepare this concept, relevant stakeholders in professional practice, health and occupational policy-making, employers, healthcare management, and patients will be surveyed in advance and involved in the development. Models and recommendations for the topics of an interprofessional competency profile are available in various other concepts and papers (WHO [24], CanMEDs [11], CIHC [25]). These could serve as a basis.

The concept to be developed and approved should also be designed so that its implementation in the legal frameworks and curricula of the separate health professions can be provided for. In addition, the concept shall be a guideline for the teaching qualification of all instructors in the health professions who are active in the area of IPE.

#### 2. Curriculum design and teaching concepts

Interprofessional teaching and learning philosophies require a curricular design that allows for systematic and targeted competence development in this area. Interprofessionalism and the ability to engage in interprofessional work should be the guiding principle. These guiding principles should affect the structures, organizational culture, and the teaching/learning culture. The necessary competencies should be described in terms of different levels of qualification (see *Europäischer Qualifikationsrahmen* [EQR] [26]). In addition to the curricular content and pedagogical approach, the structures, organizational culture and attitudes, which follow these principles, must be recognizable in the curricular design.

The committee recommends:

Educational institutions that wish to develop Interprofessional Education concepts need to have the appropriate staff and material resources, so that these concepts can take shape as an overall approach which can then be seen and recognized in different aspects, rather than simply as concepts in isolated modules and the training of individual instructors. The development of modern and innovative teaching approaches not only requires appropriate qualification of teaching staff (see also Recommendation 3), but also a corresponding assessment of capacities within the university faculties or specialty departments. Any necessary legal bases must be identified and clarified.

The focus on competency, problem-solving, situations and actions should be addressed by the pedagogical approaches and contain, among other things, the discourse surrounding case-based learning in common curricular units, common interprofessional practice and reflective phases during the practical education phases, simulations on topics such as team conferences, patient admission/discharge management, dealing with family members, as well as enquiry-based learning. Interprofessional teaching concepts must be developed and implemented by an interprofessional team. To accomplish this, it will be necessary to identify common competency areas and common curricular content and to use these for Interprofessional Education (e.g. health topics, communication, teamwork, work settings, etc.).

##### 3. Concepts for university instructor training in interprofessional teaching and learning in healthcare

The development of modern and innovative teaching and learning concepts for interprofessional learning requires appropriate teacher qualification. Presently, this is still assigned a subordinate role in teacher education, since teacher training in the health professions has still not been professionalized. Advanced training programs for teaching in higher education address relevant aspects from a pedagogical perspective, but still give little attention to the topic of interprofessional and interdisciplinary education. 

The committee recommends:

Qualification of teachers in the field of health must be supported by suitable advanced training and degree programs in vocational education / medical education / health pedagogy. There is not only a need for a better basic understanding of the necessity of training university instructors, but also for suitable concepts in education. In addition, discussions about teaching methodology in particular disciplines and interprofessional learning must be steadily intensified and structurally anchored by professorships for Interprofessional Teaching at the universities.

##### 4. Organizational development (university/departmental organization)

To implement the overall concept for Interprofessional Education, a series of measures at the institutional level (medical faculties/educational institutions) are necessary, which must indicate sustainability in their evaluations. The complexity of the stakeholders involved at the institutions varies depending on the rank of the organizational unit.

The committee recommends:

Structures must be created at the institutions that make it possible to develop, reflect on, and design interprofessional concepts. The topic of interprofessionalism must be taken up by study commissions at universities and be represented at other institutions on the relevant committees there. Working structures must be established and integrated in the institutions that follow through with and evaluate the development and implementation of IPE in the curricula. The necessary funding and organizational flexibility must be ensured. This includes taking up the topic of interprofessionalism in course evaluations as part of quality assurance and verifying the quality of the implementation. To emphasize this and ensure implementation, interprofessional learning should be added to the criteria for accreditation of health-related degree programs and training.

##### 5. Quality assurance and evaluation 

Basic structural frameworks form a crucial basis for quality assurance. Systematically developed concepts for interprofessionalism require continual evaluation regarding the quality of their process, outcome and structure in order to undertake any needed changes. Decisions about any modifications must be made by committees, for which sufficient funding has been provided.

The committee recommends:

Interprofessional Education programs that aim to realize the above conceptual elements will structurally need to have education take place at a particular site (location). This may possibly require new institutional forms and structures and even its own organizational units within the educational institutions to allow the establishment of interprofessional learning groups over a longer period of time for joint work on or treatment of a case, problem or situation. This also assumes the ability to assess interprofessional competence. To do this, the simultaneous development of suitable measuring instruments will be necessary.

##### 6. Setting up research structures in the area of Interprofessional Education and competence development

The need for research within the scope of Interprofessional Education and competence development is considerable. No substantiated knowledge about the effects of interprofessional learning situations exists yet for students of the various health professions in the German-speaking countries. Furthermore, there is a lack of knowledge regarding development parameters for professional and interprofessional identity; no approaches for vocational teaching to train interprofessional competencies exist. Relevant links to professional practice have not been identified and discussions surrounding the sociology of professions from an interprofessional perspective must be continued. Theoretical and practical requirements for the competence profile of interprofessional teachers must be defined. Moreover, it is just as important to take into consideration any benefits for the patient and the healthcare system associated with such a development.

The committee recommends: 

The creation of structures in Interprofessional Education must be accompanied by the establishment of research structures in the area of Interprofessional Education and competence development. Future research projects on the issues mentioned above need to be initiated not just by the universities, but must also be more strongly supported and initiated by professional associations and scientific societies. Targeted funding must also be provided by the federal government and states or cantons. A wider awareness for the topic also in terms of additional sources of funding is desired. A minimum requirement for relevant projects is the participation of at least two occupational groups, not only on the research team, but also in the group targeted for investigation.

##### 7. Network structures

The further development of concepts and innovations is made possible by the exchange of information and ideas at the professional level among colleagues. Up until now this exchange about aspects and issues in Interprofessional Education and Collaboration has taken place on the margins of conferences held by individual associations and professional societies (*Gesellschaft für Medizinische Ausbildung* (GMA); https://gesellschaft-medizinische-ausbildung.org/, *Hochschulverband Gesundheitsfachberufe e.V*. (HVG); http://www.hv-gesundheitsfachberufe.de/, *Deutsche Gesellschaft für Rehabilitationswissenschaften e.V*. (DGRW); http://www.dgrw-online.de/, *Deutsche Gesellschaft für Allgemeinmedizin und Familienmedizin* (DEGAM); http://www.degam.de/). IPE and IPC are hardly at the focus of such exchanges. A network within the existing structures (within and external to the university) does not exist, so that the experiences gained from the projects and models cannot be sustainably consolidated and passed on.

The committee recommends:

Network structures ensuring an exchange between institutions must be encouraged and promoted. These allow not only for the sharing of experiences among existing occupational and educational models, but also grants other interested institutions and committees the option of informing themselves about Interprofessional Education models and identifies contacts who have gathered knowledge and experience in this area. An initial starting point in respect to this is the GMA committee on Interprofessional Education in the Health Professions, which offers an opportunity for exchange. Regular conferences that focus on Interprofessional Education and Collaboration should be given financial support and promoted by the professional associations and societies.

## Strategies for implementation

To implement these recommendations, the committee on Interprofessional Education in the Health Professions views the strategies listed below as necessary. These are aimed at the various stakeholders in the fields of health and education.

1. So that the educational programs in the health professions meet the future demands placed on the healthcare system, Interprofessional Education sequences must become part of the curricula. The recommendations and strategies given here on the content and structures can contribute significantly to this. It is desired that these discussions will be further supported and followed by university administrations, medical schools, professional associations, scientific societies, and policy-makers in health and education.
2. To establish functioning interprofessional working structures, academic degree programs should be included in the health professions in terms of content and organization, in addition to the current university offerings, some already offered at the university medical schools [http://www.mft-online.de/files/140213_stellungnahme_akademisierung_pflegeberufe.pdf, last verified on 2 March 2015].
3. This position paper is meant to be taken up in the discussions with the *Medizinische Fakultätentag*, the *Verband der Universitätsklinika* (VUD), and the *Verband der Pflegedirektorinnen und Pflegedirektoren* (VPU). Furthermore, work must be done towards concrete implementation of these recommendations. The committee is available for further information and more detailed elucidation.
4. The topic of interprofessionalism has to be addressed in a sustained fashion by all committees which are in charge of the education and representation of the health professions in education and practice. This may require additional funding that would need to be provided by the federal government and state.
5. To implement the recommendations in this paper, the support of scientific projects in Interprofessional Education and their publication can be helpful, as are both recognition in the form of awards for realizing interprofessional education projects and joint organization of conferences on the topic by public institutions (among others *Bundesministerium für Bildung und Forschung* [BMBF]).

## Summary/Outlook

It has been shown that the topic of interprofessionalism in educational programs for the health professions (medicine, nursing, therapy, diagnostics etc.) is increasingly the theoretical and practical focus of discussions among experts in Germany, Austria and Switzerland. There is extensive consensus regarding recognition of the need for closer cooperation among the professions in both theory and practice. This is intended to begin early so that pupils and students can learn from, with, and alongside each other.

Problems with implementing Interprofessional Education have primarily been identified as organizational and system-related. As a result, future structures that facilitate and promote common learning and Interprofessional Collaboration must be created in the university faculties and educational institutions. This will prepare future healthcare workers well for the challenges that will face the healthcare system. Not to be overlooked is that, along with the structural aspects, there are also deficits in the attitudes and cultures of the pre- and postgraduate groups of health professionals, which must be worked on. 

To ensure the sustainability of current and future projects concerning Interprofessional Education, it is important to first develop a suitable overall concept that takes all aspects into consideration. In addition, organizational and political structures must be established and resources must be secured to make the well-founded development of Interprofessional Education programs possible. The programs are to be integrated in terms of curriculum, continually evaluated and developed further. In parallel, structures must be created and funding guaranteed in order to make appropriate research on Interprofessional Education and Collaboration possible. This should include the effect of Interprofessional Education concepts and approaches on the students and teachers, as well as on the provision of healthcare. The development of quality assurance instruments must also occur.

As this is being accomplished, the committee for Interprofessional Education in the Health Professions can assume the function of a network. It is also important to expand suitable structures for Interprofessional Education in the scientific societies in order to compile knowledge and experience in the future and to ensure professional representation of interprofessional concerns in education and practice.

## Notes

The position paper was accepted by the GMA executive board on 30-01-2015.

^1^ The term “health professions” in the English version of the position statement denotes all the health professions including medicine, nursing, therapy and diagnostics., etc. In contrast, in the German language (and therefore in the German version of the position statement), the terms *Gesundheitsberufe* and *Gesundheitsfachberufe* do not typically mean the medical profession. Also, important to note is that in the German-speaking countries nursing, therapy professions and midwifery are currently undergoing a transition process from vocational training (usually hospital-based) to university-based education, which we signify with the term “academization”. In Switzerland and Austria, the occupations in diagnostics, are also experiencing academization.

^2^ German-speaking countries: Germany – Austria – Switzerland

## Competing interests

The authors declare that they have no competing interests.
